# Anterior Cervical Discectomy and Fusion Using Zero-P System for Treatment of Cervical Spondylosis: A Meta-Analysis

**DOI:** 10.1155/2021/3960553

**Published:** 2021-12-16

**Authors:** Zhaoyang Guo, Xiaolin Wu, Shuai Yang, Chang Liu, Youfu Zhu, Nana Shen, Zhu Guo, Weiliang Su, Yan Wang, Bohua Chen, Hongfei Xiang

**Affiliations:** ^1^Department of Orthopedics, Affiliated Hospital of Qingdao University, Qingdao 266003, China; ^2^Department of Rehabilitation, Affiliated Hospital of Qingdao University, Qingdao 266003, China

## Abstract

**Objective:**

The current study aimed to explore the efficacy of Zero profile intervertebral fusion system (Zero-P) and traditional anterior plate cage system (PC) in the treatment of cervical spondylotic myelopathy (CSM). Further, the present study evaluated effects of the treatments on medical security, height of intervertebral disc, adjacent-level ossification development (ALOD), and adjacent segmentation disease (ASD) through a systematic retrospective analysis.

**Methods:**

Studies on Zero-P system and traditional anterior plate cage system for ACDF in the treatment of CSM were searched in PubMed, Web of Science, Ovid, Embase, and Cochrane Library databases. Two independent researchers screened articles, extracted data, and evaluated the quality of the articles based on the inclusion and exclusion criteria of the current study. RevMan5.3 software was used for meta-analysis following the guidelines of Cochrane collaboration network. Cervical curvature, interbody fusion rate, preoperative and postoperative disc height index (DHI), fusion cage sinking rate, postoperative dysphagia, ASD, ALOD, and loosening of screw were compared between the two groups.

**Results:**

A total of 17 literatures were included in the present study, including 6 randomized controlled trials and 11 observational studies. The studies comprised a total of 1204 patients with CSM, including 605 patients in the Zero-P system group (Zero-P group) and 599 patients in the traditional animal plate cage group (PC group). Results of this meta-analysis showed that postoperative dysphagia [OR = 0.40, CI (0.28, 95% 0.58), *P* < 0.00001], ALOD [OR = 0.09, CI (0.02, 95% 0.39), *P* = 0.001], ASD [OR = 0.42, CI (0.20, 95% 0.86), *P* = 0.02], and screw loosening [OR = 0.20, CI (0.08, 95% 0.52), *P* = 0.0009] of the Zero-P group were significantly lower compared with the PC group. On the other hand, preoperative cervical curvature [WMD = −0.23, CI (−1.38, 95% 0.92), *P* = 0.69], postoperative cervical curvature [WMD = −0.38, CI (−1.77, 95% 1.01), *P* = 0.59], cage sinking rate [OR = 1.41, CI [0.52, 95% 3.82], *P* = 0.50], intervertebral fusion rate [OR = 0.76, CI (0.27, 95% 2.48), *P* = 0.38], preoperative DHI [WMD = −0.04, CI (−0.14, 95% 0.22), *P* = 0.65], and postoperative DHI [WMD = 0.06, CI (−0.22, 95% 0.34), *P* = 0.675] were not significantly different between the two groups.

**Conclusion:**

It was evident that the Zero-P system used in ACDF is superior compared with the traditional anterior plate cage system in postoperative dysphagia, avoiding ALOD, ASD, and screw loosening.

## 1. Introduction

Incidence of degenerative diseases is annually increasing due to the increase in the number of elderly population. Therefore, previous studies have also shown an increase in the incidence of cervical spondylotic myelopathy (CSM) which is a common cause of spinal cord dysfunction. Currently, the onset of CSM occurs at an early age and the condition is becoming more complicated. Several studies have explored methods for effective alleviation of spinal cord compression in patients with CSM and restoration of the spinal cord function. When conservative methods are ineffective or in the case of worsening symptoms, active surgical treatment is recommended for patients with CSM to release nerve compression for timely restoration of normal spinal cord function [1, 2]. The commonly used cervical spine anterior approaches for surgical treatment of cervical spine diseases are cervical spine posterior approach, combined anterior, and posterior surgery as well as the various minimally invasive techniques.

Anterior cervical surgery was first reported as a safe and effective method for treatment of the degenerative cervical spondylosis by Cloard, Smith, and Robinson in 1958. Anterior cervical discectomy and fusion (ACDF) surgery is one of the most advanced cervical spine surgery approaches which play an important role in treatment of cervical disease [3–5]. Anterior cervical discectomy and fusion (ACDF) is conventionally fixed with anterior interbody fusion cage and steel plate. This fixing system has several advantages but is also associated with potential disadvantages. The most common shortcomings of these techniques include fracture or loosening of plates and screws, tracheal-esophageal interference and influence, and difficulties in postoperative swallowing [6, 7].

Recent studies have explored a lower, more smoothly contoured Zero-P system that reduces incidence of dysphagia after ACDF. Notably, the system can be fully implanted in the intervertebral space, providing sufficient stability and avoiding contact between the implant and the prevertebral soft tissue [8, 9]. Therefore, the zero-notch interbody fusion and internal fixation system is widely used in ACDF to reduce occurrence of these complications.

Currently, it is not clear whether the Zero-P system significantly reduces the incidence of postoperative ALOD, ASD, and screw loosening compared with the traditional anterior plate cage system. Therefore, the aim of the current meta-analysis was to summarize the available evidence from high-quality relevant studies and explore the effects of using Zero-P system as well as traditional anterior plate cage system. The findings of this study can help in clinical decision-making.

## 2. Materials and Methods

### 2.1. Search Strategy

English articles were retrieved for this study from PubMed, Web of Science, Ovid, Embase, and Cochrane Library databases. Literature search was carried out based on the search terms determined by the PICOS principle. The present study included English articles about studies on the use of Zero-P and titanium plate combined with cage for ACDF surgery from the time of inception of the databases to December, 2020. Clinical studies on efficacy of treatment degenerative cervical spondylosis were selected using the following keywords and phrases: “Zero-P,” “Zero Profile,” “anterior cervical discectomy and fusion,” and “ACDF” as search terms. The keywords were searched independently and all synonyms as well as variants of the keywords were searched by combining free words and subject words concurrently. Free words and subject words of each keyword were searched by the logical connection word “OR,” and the logical connection word “AND.” The search group segment was connected and the search results were retrieved.

### 2.2. Literature Screening and Data Extraction

Inclusion criteria for the present study were as follows: (1) research type: randomized controlled trial and observational study; (2) research object: cervical spondylotic myelopathy; (3) intervention measures: the experimental group represents the Zero-P group, and the control group was the titanium plate cage group (PC Group); (4) follow-up time: 12 months or more; (5) comparative data: ① imaging parameters including preoperative and postoperative follow-up cervical spine curvature, degree of intervertebral fusion, and preoperative intervertebral height index (DHI); ② complications including postoperative dysphagia, cage sinking, and adjacent segment ossification (ALOD); adjacent segment disease (ASD); and screw migration (screw migration), and the literature should have at least one outcome indicator.

Exclusion criteria for this study included the following: ① only studies on Zero-P or titanium plate cage; ② reviews, conference papers, abstracts, or unpublished documents; ③ incomplete data or documents with errors that may affect results; ④ repeated papers; ⑤ research design for self-comparison before and after or without a control group; ⑥ studies with trial design which is not rigorous or inappropriate statistical methods; ⑦ other types of zero-notch interbody fusion internal fixation systems, such as PREVALIL; and ⑧ follow-up time less than 12 months.

Titles and abstracts of the articles retrieved based on inclusion and exclusion criteria were read. Articles that did not meet the inclusion criteria were excluded after reading the title and abstract. Full texts of the documents that met the inclusion criteria were then read to further explore whether they met the inclusion criteria. The original author was contacted whenever the original data was found to be unclear. Two reviewers carried out independent data extraction for articles that met the including criteria. The two reviewers jointly developed a standard data extraction table and, after data extraction, each reviewer cross-checked the data for their partner. Any disagreement between the reviewers was resolved by a third reviewer.

### 2.3. Methodological Quality Evaluation

Randomized controls were compared from seven aspects including random sequence generation, allocation hiding, double blinding of participants and staff, blinding of result evaluation, data completeness, selective outcome reports, and other sources of bias following the evaluation criteria of the Cochrane Evaluation Manual. The quality of included observational studies was evaluated using Newcastle-Ottawa Scale (NOS). Evaluation was independently conducted and cross-checked by two researchers. However, any case of disagreement was resolved through a third evaluator.

### 2.4. Statistical Analysis

Statistical analysis of the data obtained in the present study was performed using Review Manager 5.3 (RevMan5.3) software which was developed by Cochrane collaboration network. Analysis of continuous variables in the current study including cervical vertebra Cobb angle, preoperative, and postoperative DHI was carried out using weighted mean difference (WMD) at 95% confidence interval (95% CI). Odds ratio (OR) and 95% CI were used for analysis of association between the continuous variables of the current study and adjacent segmental ossification rate, adjacent segmental disease incidence, dysphagia incidence, interbody fusion rate, fusion cage sinking rate, and screw loosening rate. Statistically significant difference was set at *P* < 0.05.

**Figure 1:**
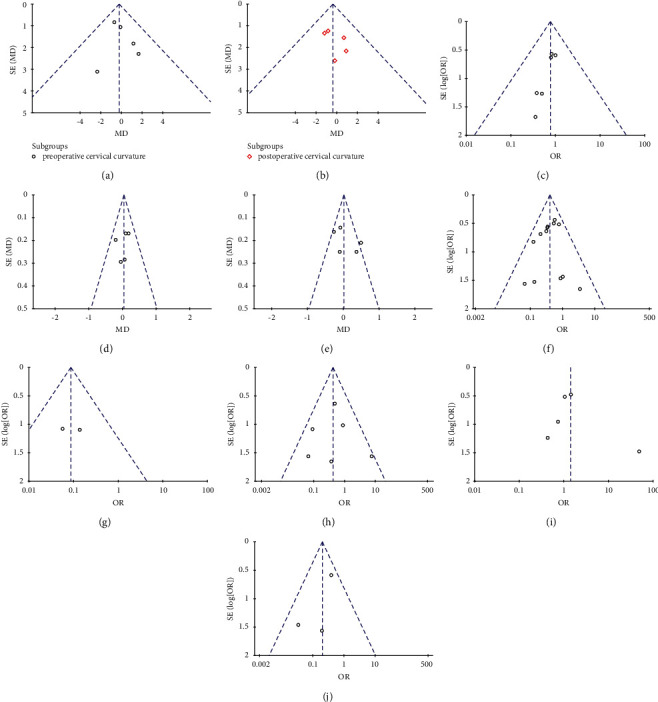
(a) Preoperative cervical curvature funnel diagram; (b) postoperative cervical curvature funnel diagram; (c) funnel diagram for interbody fusion rate; (d) preoperative DHI funnel diagram; (e) postoperative DHI funnel diagram; (f) postoperative dysphagia funnel diagram; (g) ALOD funnel diagram; (h) ASD funnel diagram; (i) postoperative sinking rate funnel diagram of fusion cage; (j) screw loosening funnel diagram (IV) evaluation of publication bias.

Chi-square and *I*^2^ tests were used to evaluate the heterogeneity of the included studies. A *P* > 0.1 for the chi-square test and *I*^2^ < 50% implied that the heterogeneity was low. Fixed effect model was used for determining the combined effect. When the heterogeneity was high, individual studies were singly eliminated for sensitivity analysis to find the source of heterogeneity. Funnel charts were generated to determine the publication bias for studies comprising more than ten articles (Figure 1).

## 3. Results

### 3.1. Search Results

**Figure 2:**
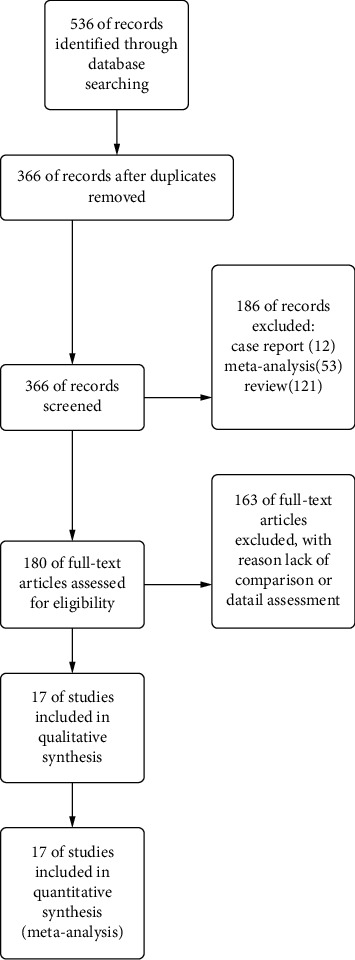
Flowchart of literature screening.

A total of 536 studies were obtained from the databases following an independent search conducted by two scholars. The search was conducted according to the predesigned retrieval strategy. A total of 480 articles were obtained after eliminating cross-documents and repeated published documents. Among the 480 articles, a total of 72 articles were obtained after excluding documents that did not meet the inclusion criteria. A total of 55 abstracts and full papers were then excluded based on the inclusion and exclusion criteria. Finally, it was found that 17 studies met the inclusion criteria and were hence included in the current study (Figure 2 and Table 1).

### 3.2. Quality Evaluation

**Figure 3:**
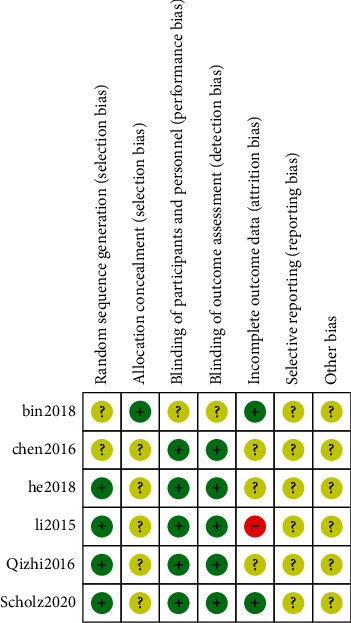
Literature quality evaluation chart of RCT.

Six of the 17 original studies included in the present study [10–15] were randomized controlled trials whereas 11 studies were observational studies [16–26]. Randomized controlled studies were evaluated based on the evaluation criteria of the Cochrane evaluation manual including 7 items (Figure 3). Newcastle-Ottawa Scale was used to evaluate the quality of observational studies. Further, 9, 6, and 2 studies were allocated 7, 8, and 9 stars, respectively, implying that the studies were of high quality (Figure 3 and Table 2).

### 3.3. Meta-Analysis

#### 3.3.1. Imaging Parameters

*(1) Cervical Curvature*. It was found that a total of 5 studies including 1 randomized controlled trial and 4 nonrandomized retrospective studies [15, 17, 22, 25, 26] reported C2-C7 cervical spine curvature before and after surgery (Table 3).

**Figure 4:**
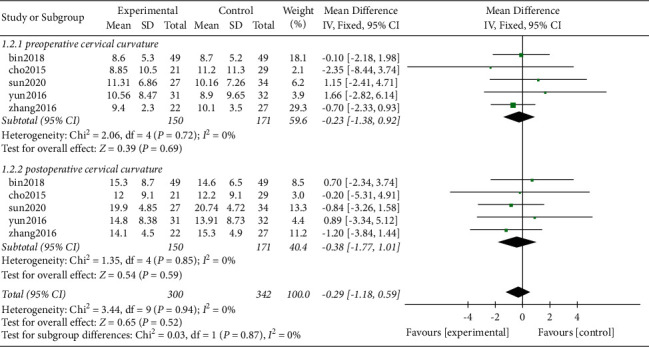
Cervical curvature before and after surgery.

A total of 321 patients were included based on preoperative cervical curvature as the evaluation index. Out of the 321 patients, 150 of them were in the Zero-P group whereas 171 patients were in the PC group. All the studies passed the heterogeneity test (*P* = 0.72, *I*^2^ = 0% for each). Analysis results of this study showed that there was no heterogeneity between the original studies. In addition, the fixed effects model was used for analysis of combined effect size of the preoperative C2-C7 cervical spine curvature between the two groups (WMD = −0.23, 95% CI [−1.38, 0.92], *P* = 0.69). The finding of this study showed that the difference was not statistically significant (Figure 4).

A total of 321 patients were included in the present study, out of which 150 patients were in the Zero-P group whereas 171 patients were in the PC group for evaluation based on postoperative cervical curvature. Moreover, the studies passed the heterogeneity test (*P* = 0.85, *I*^2^ = 0%; each). These findings evidently show that there was no heterogeneity between the original studies. The fixed effect model was used for analysis of combined effect size of C2-C7 cervical spine curvature and the findings showed that there was no significant difference between the two groups (WMD = −0.38, 95% CI[−1.77, 1.01], *P* = 0.59, Figure 4).

*(2) Intervertebral Fusion Rate*. A total of 6 original studies including 2 randomized controlled trials and 4 nonrandomized retrospective studies [13, 14, 17, 18, 22, 25] reported fusion rate and provided valid data. A total of 383 patients were included in these studies, including 184 and 199 patients in the Zero-P group and PC groups, respectively.

**Figure 5:**
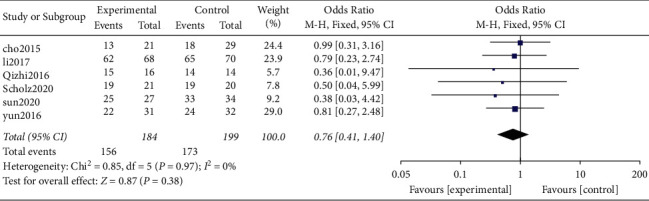
Intervertebral fusion rate.

Results of heterogeneity analysis showed that there was no heterogeneity between the original studies (*P* = 0.97, *I*^2^ = 0%). Further, the analysis of combined effect size using fixed-effects model showed that the difference between the two groups was not statistically significant (OR = 0.76, 95% CI [0.27, 2.48], *P* = 0.38, Figure 5).

**Figure 6:**
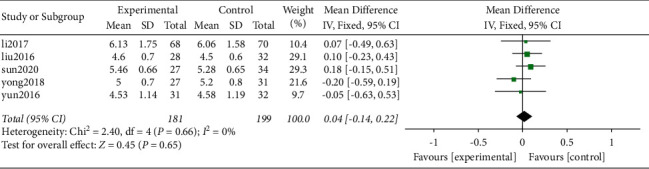
Preoperative DHI.

*(3) Disc Height Index (DHI)*. It was found that a total of 5 original studies [18–20, 22, 25] reported the intervertebral height index and provided valid data. All the 5 studies were nonrandomized retrospective studies. A total of 380 cases including 181 and 199 in the Zero-P group and PC groups, respectively, were included based on preoperative intervertebral height index. The results of heterogeneity analysis showed no heterogeneity between the original studies (*P* = 0.66, *I*^2^ = 0%). Further, the analysis of the combined effect size using fixed-effect model showed no statistically significant difference in preoperative intervertebral height index between the two groups (WMD = −0.04, 95% CI [−0.14, 0.22], *P* = 0.65; Figure 6).

**Figure 7:**
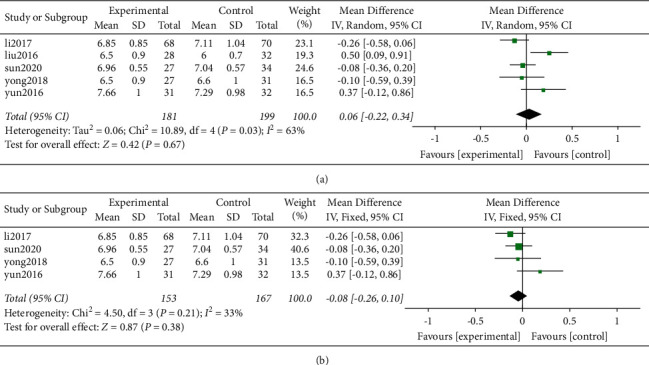
(a) Postoperative DHI and (b) sensitivity analysis on postoperative DHI.

A total of 380 cases including 181 and 199 in the Zero-P group and PC groups, respectively, were selected based on the postoperative intervertebral height index. Results of heterogeneity analysis in the current study showed a high heterogeneity between the original studies (*P* = 0.03, *I*^2^ = 63%). Analysis of the combined effect using the random effects model showed no significant difference in intervertebral height index between the two groups (WMD = 0.06, 95% CI [−0.22, 0.34], *P* = 0.675; Figure 7(a)). Sensitivity analysis was carried out by eliminating individual studies one by one.

Removal of a study by Liu (2016) significantly decreased heterogeneity (*P* = 0.21, *I*^2^ = 33%). Further, the results of the analysis of the combined effect using the fixed effects model showed that the difference between the two groups was not statistically significant (WMD = −0.08, 95% CI [−0.26, 0.10], *P* = 0.38, Figure 7(b)).

#### 3.3.2. Postoperative Complications

*(1) Dysphagia*. It was found that a total of 13 original studies [10–16, 18, 20, 22, 23, 25, 26] reported dysphagia and provided valid data. Out of the 13 studies, 6 of them were randomized controlled trials whereas 7 were nonrandomized retrospective studies.

**Figure 8:**
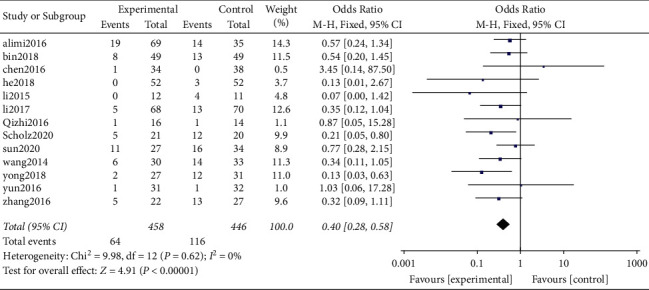
Postoperative dysphagia.

A total of 904 cases were included including 458 and 446 patients in the Zero-P group and PC groups, respectively, based on incidence of postoperative dysphagia as the evaluation criteria. Results of heterogeneity analysis showed no heterogeneity between the original studies (*P* = 0.62, *I*^2^ = 0%). On the other hand, the results of analysis of combined effect using the fixed-effect model showed a significant difference between the two groups (OR = 0.40, 95% CI [0.28, 0.58], *P* < 0.00001). The incidence of dysphagia in the Zero-P group (13.97%) was significantly lower compared with that in the PC group (26.01%; Figure 8).

**Figure 9:**
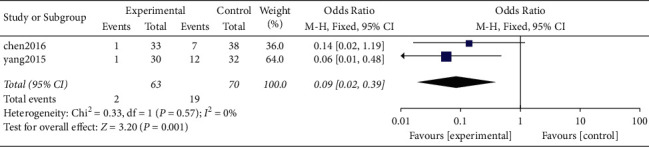
Postoperative ALOD.

*(2) Adjacent-Level Ossification Development (ALOD)*. A total of 2 original studies, including 1 randomized controlled trial and 1 nonrandomized retrospective study [10, 24], reported ALOD findings and provided valid data. Further, a total of 133 cases were included, including 63 and 70 patients in the Zero-P group and PC groups, respectively. Heterogeneity was analyzed and the result showed no heterogeneity between the original studies (*P* = 0.57, *I*^2^ = 0%). Analysis results of the combined effect using fixed-effects model showed that the incidence of ALOD in the Zero-P group (3.17%) was significantly lower compared with that in the PC group (27.14%) (OR = 0.09, 95% CI [0.02, 0.39], *P* = 0.001; Figure 9).

**Figure 10:**
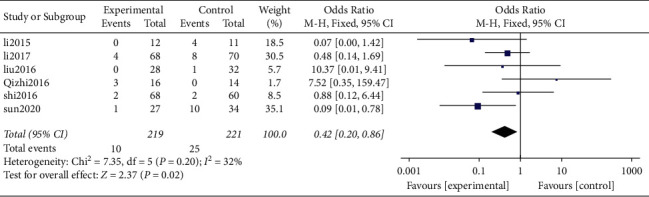
Postoperative ASD.

*(3) Adjacent Segment Disease (ASD)*. It was found that a total of 6 original studies including 2 randomized controlled trials and 4 nonrandomized retrospective studies [12, 13, 18, 19, 21, 22] reported ASD and provided valid data. A total of 440 cases were included in the present meta-analysis, including 219 and 221 patients in the Zero-P group and pc groups, respectively. Results of the heterogeneity analysis showed insignificant heterogeneity between the original studies (*P* = 0.20, *I*^2^ = 32%). Analysis results for the combined effect using the fixed-effect model showed that the incidence of ASD in the Zero-P Group (4.57%) was significantly lower compared with that in the PC group (11.31%) (OR = 0.42, 95% CI [0.20, 0.86], *P* = 0.02; Figure 10)

**Figure 11:**
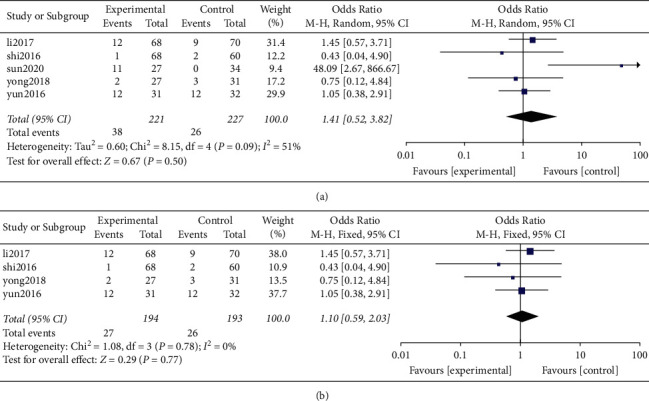
(a) Fusion sinking rate after surgery. (b) Sensitivity analysis of fusion sinking rate after operation.

*(4) Sinking Rate of the Cage*. A total of 4 original studies [18, 20, 22, 25] reported the sinking rate of the cage and provided valid data. All the 4 studies were nonrandomized retrospective studies. Notably, a total of 448 cases were based on postoperative fusion cage sinking rate including 221 and 227 patients in the Zero-P and PC groups respectively. Results of heterogeneity analysis showed a high heterogeneity among the original studies (*P* = 0.09, *I*^2^ = 51%). The results of the combined effect analysis using the random effects model showed that the difference between the two groups was not statistically significant from each other (OR = 1.41, 95% CI [0.52, 3.82], *P* = 0.50; Figure 11(a)).

Sensitivity analysis was carried out by eliminating individual studies one by one. It was found that the removal of a study by Sun (2020) significantly reduced the heterogeneity (*P* = 0.78, *I*^2^ = 0%). It was also found that the combined effect analysis using the fixed effects model showed no statistical difference between the two groups (OR = 1.10, 95% CI [0.59, 2.03], *P* = 0.77; Figure 11(b)).

*(5) Screw Loosening*. Results of the present study show that a total of 3 original studies [18, 19, 21] had screw loosening and provided valid data. Further, all the 3 studies were nonrandomized retrospective studies.

**Figure 12:**
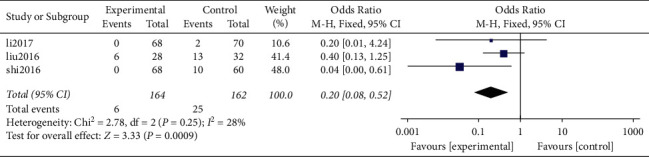
Screw loosening after operation.

A total of 326 patients were included based on postoperative screw loosening, including 164 of them in the Zero-P group and 162 patients in the PC group. Heterogeneity analysis showed low heterogeneity between the original studies (*P* = 0.25, *I*^2^ = 28%). The results of combined effect analysis of the fixed-effect model showed significant difference between the two groups (OR = 0.20, 95% CI [0.08, 0.52], *P* = 0.0009). Incidence of screw loosening in the Zero-P group (3.66%) was significantly lower compared with that in the PC group (15.43%) (Figure 12).

## 4. Discussion

Anterior cervical discectomy and bone graft fusion (ACDF) is a safe and an effective surgical method for the treatment of degenerative cervical spine diseases [27]. Anterior titanium plate cage is used in ACDF and has become a conventional surgical method for treatment of degenerative cervical spondylosis [28]. It significantly restores the height of intervertebral disc of spine, ensures high bone graft fusion rate, preserves segmental lordosis, and has strong corner ability [28–30]. However, the titanium plate is associated with several limitations, such as screw loosening, titanium plate displacement, soft tissue injury, adjacent segment disease, adjacent segment ossification, and increased incidence of dysphagia [8, 31, 32].

Therefore, Zero-P interbody fusion cage was developed to circumvent limitations of the titanium plate. It is a cervical fusion system that can be independently used in single-segment or multisegment anterior degenerative cervical spondylosis [33]. Several previous studies have reported that Zero-P interbody fusion cage significantly limits the potential risks of dysphagia after fixed surgery of cervical vertebrae, which is in agreement with the findings of the current study [34]. However, there has been no systematic review and analysis conducted to compare the effects of the two techniques on cervical spine curvature, intervertebral height, ALOD, and ASD.

### 4.1. Zero-P Significantly Reduces Incidence of Long-Term ASD Compared with Traditional Anterior Steel Plates

Anterior cervical discectomy and fusion method is associated with high incidence of ASD. In addition, the traditional fixation methods cause ASD, which may eventually require additional treatment [35–37]. The exact pathophysiological mechanism of ASD has not been fully explored [35–41]. It may be derived from the existing lesions in adjacent segments and changes in biomechanical forces near the previous fusion site and this may increase the risk of degenerative changes [42]. Previous studies have shown that the biomechanical changes of adjacent vertebral bodies after spinal fusion are the major causes of ASD.

Cunningham et al. [43] used a specially designed pressure needle transducer to quantify the intradiscal pressure changes at the level of 3 adjacent intervertebral discs in 11 patients. The findings of that study showed that the proximal disc pressure increased by 45% in case of instability and internal fixation of the fusion zone. It was also found that the presence of steel plates may increase risk of degenerative changes in adjacent segments. Several previous studies have also reported the range of motion and intradiscal pressure increase in untreated segments adjacent to the fused segment [37, 40, 41, 44, 45]. According to Hilibrand and Robbins [44] approximately 25% of patients who used traditional steel plates for single-segment ACDF treatment developed ASD within 10 years.

Previous studies on the effect of Zero-P internal fixation system in reducing occurrence of long-term ASD reported inconsistent findings. A study conducted on 71 patients by Chen et al. [46] reported that there was no significant difference in incidence of degenerative diseases in the adjacent segments after treatment with Zero-P and plate cage. In addition, in a separate study, a total of 79 patients with cervical spondylopathy were also treated with anterior cervical fusion and internal fixation. Out of the 79 patients, 41 of them were in the Zero-P group whereas 38 patients were in the steel cage internal fixation group. Incidence of ASD in the two groups was 14.63 and 26.31%, respectively. These findings show that the Zero-P device was more effective in reducing degeneration of adjacent segments after degenerative cervical disease compared with the plate cage internal fixation system. However, it was found that the difference between the two groups was not statistically significant. It is, hence, not clear whether the use of Zero-P system treatment reduces incidence of postoperative ASD.

In the current study, the total number of patients with cervical spondylopathy included was 440, with 219 and 221 of them in the Zero-P and PC groups, respectively. The findings of this study showed that the incidence of ASD in the Zero-P group (4.57%) was significantly lower compared with that in the PC group (11.31%). Higher efficacy may be because Zero-P fixes the intervertebral disc space away from the adjacent segment, thus reducing the impact on the biomechanics of the adjacent segment. Therefore, it is evident that Zero-P minimizes the risk of degeneration of adjacent intervertebral discs.

### 4.2. Zero-P Reduces Incidence of Long-Term ALOD Compared with Traditional Anterior Steel Plates

Adjacent-level ossification development (ALOD) is a common complication of ACDF which occurs as early as 3 months after surgery [47]. Previous studies have shown that cervical spine plate is associated with increased risk of ALOD. According to Garrido et al. [48], the incidence of ALOD in cervical disc replacement during two-year and four-year follow-up was significantly lower compared with that of plate fixation. In a separate study, Yang et al. [24] performed a retrospective study and reported that Zero-P was associated with lower incidence of ALOD. In addition, the length of the steel plate was associated with the incidence of ALOD. Further, Park et al. [35] explored the incidence of ALOD after internal fixation of the anterior cervical plate. The findings of the study showed that the incidence of ALOD was higher when the distance between the tip of the plate and the adjacent intervertebral disc was less than 5 mm. According to Lee et al. [49] and Park et al. [50], the use of short plates with inclined screw tracks reduces occurrence of ALOD.

Findings of the current study showed that the incidence of ALOD in the Zero-P group (3.17%) was significantly lower compared with that of the PC group (27.14%), which were in agreement with findings from previous studies. Although the anterior longitudinal ligament was injured by a spreader or an electric knife in the two groups of patients, it was evident that the plate promoted formation of osteophytes during repair of the anterior longitudinal ligament. On the other hand, the Zero-P group had no plate internal fixation and no mechanical stimulation; hence, the incidence of ALOD was relatively low.

### 4.3. Zero-P Reduces Incidence of Dysphagia Compared with Traditional Anterior Plates

Dysphagia is a complication of ACDF after using additional anterior plate. Previous studies have reported that the incidence of postoperative dysphagia is as high as 71%. Incidence of persistent dysphagia can reach 35.1% after 7.2 years of anterior cervical plate fixation, but most symptoms of dysphagia decrease within a month. However, between 12 and 14% of patients presented with difficulties in swallowing 1 year after surgery [51]. Possible causes of dysphagia include postoperative soft tissue edema, esophageal injury, postoperative hematoma, and adhesions around the implanted cervical spine plate. Moreover, the anterior cervical plate is placed directly behind the esophagus, which may affect or irritate the esophagus. Previous studies have reported that the design and thickness of the anterior locking plate are correlated with postoperative dysphagia. According to Lee et al. [34], a correlation between plate thickness and incidence of dysphagia was reported, and thus the use of thinner plates can reduce incidence of dysphagia. Another possible mechanism of dysphagia after ACDF anterior plate surgery may be the need for additional traction to place the anterior locking plate. During the process of anterior plate implantation, it has been reported that an increase in esophageal pressure may cause dysphagia in patients with ACDF anterior plate. Furthermore, the Zero-P cervical fusion cage does not straddle the anterior vertebral body and can be completely contained in the decompressed intervertebral space. Therefore, there is a reduced mechanical stimulation of esophagus and other prevertebral soft tissues, and it retains as much normal anatomy as possible. This explains the lower incidence of postoperative dysphagia in the Zero-P group.

### 4.4. Zero-P and Traditional Anterior Plate Show No Significant Difference in Maintenance of Cervical Spine Curvature and Intervertebral Height

The curvature of the cervical spine plays an important role in maintaining efficacy of surgery. Poor cervical spine curvature increases stress distribution of the internal fixation device and adjacent segments, thus increasing the incidence of internal fixation failure and ASD. It has been reported that insufficient recovery of cervical spine curvature after ACDF significantly affects cervical spine instability and postoperative axial pain and may also affect the recovery of nerve function [52]. However, the role of Zero-P in maintaining postoperative cervical spine curvature is controversial. According to Shi et al. [53], the loss of cervical spine curvature in the Zero-P group was significantly higher compared with that in the PC group after a 30-month follow-up.

A study by Chen et al. [46] reported that the average C2-C7 Cobb angle of the traditional steel plate group was significantly greater compared with that of the Zero-P group. Use of steel plate can also reconstruct the ideal sagittal position balance with the spine compared with Zero-P fixation. A separate study by Lan et al. [54] reported that the cervical spine Cobb angle was significantly corrected after the operation in the Zero-P group and the traditional plate group with no statistical difference, which is in consonance with the findings of the current study. The findings of the current meta-analysis study showed that there was no statistical difference between the two groups; however, postoperative cervical spine curvature was significantly improved as compared to preoperative cervical spine curvature.

Furthermore, a drop in intervertebral height caused by sinking of the cage is a common postoperative complication of ACDF [25]. It is defined as the loss of more than 2 mm of disc height in two measurements [6]. Previous studies have shown that sinking of the fusion cage is associated with several factors including preoperative cervical spine curvature, size of the plate, contact area with the endplate, age, and the titanium plate as well as the distance between the implant and the anterior edge of the vertebral body [33, 55–57].

The findings of a study by Wu et al. [58] showed that a decrease in the height of the intervertebral disc was related to sinking of the intervertebral fusion cage. Notably, Zero-P interbody fusion cage can effectively restore the physiological structure of the cervical spine and maintain the height of the intervertebral space more effectively compared with traditional steel plates. Results of a different study by Lee et al. [59] revealed that the sinking rate of the Zero-P device (21.7%) was higher compared with that of the front steel plate (11.1%). On the other hand, Scholz et al. [60] reported that during the 6-month follow-up, the patients treated with the Zero-P device did not present sinking of the intervertebral fusion cage. According to Noh and Zhang [61] the settlement rate of Zero-P group (25%) was slightly higher at the last follow-up, compared with that of the plate cage group (21%). However, the difference between the two groups was not statistically significant from each other. In the current meta-analysis, it was found that the sinking rate of the Zero-P group was 17.19%, whereas that of the PC group was 11.45%. Although the results were not statistically different, it was evident that the sinking rate of the fusion cage in the Zero-P group was higher compared with that of the PC Group, which is consistent with the findings of a study by Noh and Zhang [61].

The findings of the present meta-analysis show that both methods can effectively maintain intervertebral height. During the operation, a distractor was used to open the intervertebral space and a Zero-P intervertebral fusion cage or a traditional steel plate cage was implanted. Therefore, the height of the intervertebral space was significantly increased compared with the space before the operation and hence restoring the intervertebral height.

### 4.5. Zero-P Reduces Incidence of Screw Loosening Compared with Traditional Front Steel Plates

According to a study by Vaccaro et al., the incidence of traditional anterior cervical fixation of plate screws and plate loosening was 15.4%, whereas the fracture rates of screws and plates were as high as 13.3 and 6.7%, respectively. Notably, plates and bone grafts were displaced (with or without transplantation). The incidence of bone fracture was high (21.4%) whereas the incidence of implant failure for long-segment plates (intervertebral screws and plates of unfused segments) ranged from 0 to 12.5%.

The design of the implant has different screw fixation mechanisms and loosening of the implant screw may be related to the design of the fixed plate-screw interface. It has been found that the “zero notch” design of the Zero-P intervertebral fusion cage has more advantages compared with the traditional plate cage system. The intervertebral screw of the Zero-P system is a self-tapping screw, which can strengthen the thread and the screw during screwing. Further, the bite force of the bone between the vertebral bodies increases the immediate stability between the vertebral bodies. Moreover, the angle of the screw and that of the cervical spine biological force line are larger compared with those for the traditional steel plate, and the pullout resistance is stronger. Therefore, the findings of the current study revealed that the incidence of screw loosening in the Zero-P group (3.66%) was significantly lower compared with that in the PC group (15.43%), which can be attributed to the described reasons.

## 5. Limitations

This study had some limitations. ① Although random effects models and sensitivity analysis were used to eliminate statistical heterogeneity, they may have led to a certain degree of measurement error. ② Although most effect sizes are sensitive, after sensitivity analysis, heterogeneity was eliminated or reduced; however, there was still some heterogeneity after the merging of individual effect data and some results could not be reliable. ③ At present, the application of Zero-P is in the early stage, the clinical practice has not been fully carried out, and the corresponding high-quality clinical research requires further long-term follow-up.

## 6. Conclusion

In conclusion, it was evident that the use of Zero-P system during anterior cervical discectomy and fusion reduces the risk of ALOD, ASD, and screw loosening and reduces the operation time, intraoperative blood loss, and the incidence of postoperative dysphagia compared with the traditional anterior plate cage system.

## Figures and Tables

**Table 1:** Quality evaluation of RCT.

First author and author (year of publication)	Country	Type of study	Surgical segment	Sample size		Gender (M/F)		Age (*x* ± *s*, years)		Follow-up time (*x* ± *s*)	
				Zero-P	PC	Zero-P	PC	Zero-P	PC	Zero-P	PC
Li et al. (2015) [12]	China	Randomized controlled trial	1	12	11	7/5	5/6	50.3 ± 8.8	51.1 ± 6.7	24	24
He et al. (2018) [11]	China	Randomized controlled trial	2	52	52	28/24	27/25	55.4 ± 12.4	59.5 ± 12.6	24	24
Yan and Nie (2018) [15]	China	Randomized controlled trial	1	49	49	29/20	29/20	43.1 ± 5.3	43.3 ± 5.2	12	12
Chen et al. (2016) [10]	China	Randomized controlled trial	3	34	38	21/13	25/13	56.9 ± 5.9	56.2 ± 5.7	12	12
Qizhi et al. (2016) [13]	China	Randomized controlled trial	2	16	14	11/5	9/5	48.13 ± 5.98	46.79 ± 5.15	32.4	32.4
Scholz et al. (2020) [14]	Germany	Randomized controlled trial	1	21	20	13/8	11/9	58	58	24	24
Alimi et al. (2016) [16]	United States	Nonrandomized retrospective study	1, 2, 3	69	35	35/34	18/17	58.2 ± 1.45	51.5 ± 1.95	15.7 ± 1.23	14.8 ± 2.13
Li et al. (2017) [18]	China	Nonrandomized retrospective study	1, 2, 3, 4	68	70	41/27	45/25	50.6 ± 7.5	51.3 ± 7.9	29.7 ± 6.5	30.8 ± 6.6
Liu et al. (2016) [19]	China	Nonrandomized retrospective study	3, 4	28	32	10/18	12/20	56.6 ± 9.7	57.5 ± 9.5	23.3 ± 6.9	24.2 ± 6.4
Cho et al. (2015) [17]	Korea	Nonrandomized retrospective study	1	21	29	12/9	19/10	56.1 ± 12	55.2 ± 10.4	24	24
Shi et al. (2016) [21]	China	Nonrandomized retrospective study	1	68	60	33/35	24/36	47.4 ± 7.0	46.5 ± 6.8	48	48
Sun et al. (2020) [22]	China	Nonrandomized retrospective study	3	27	34	15/12	25/9	54.7 ± 7.6	56.4 ± 7.5	60	60
Wang et al. (2014) [23]	China	Nonrandomized retrospective study	1, 2	30	33	18/12	14/19	56.8 ± 11.0	54.0 ± 10.0	24.1 ± 7.8	23.8 ± 8.2
Yang et al. (2015) [24]	China	Nonrandomized retrospective study	1, 2, 3	30	32	20/10	22/10	44.1 ± 5.8	42.8 ± 6.1	30.6 ± 2.4	33.1 ± 3.0
Shen et al. (2018) [20]	China	Nonrandomized retrospective study	1, 2, 3	27	31	16/11	14/17	52.3 ± 9.2	54.7 ± 9.2	37.2 ± 22.8	46.8 ± 21.6
Yun et al. (2016) [25]	Korea	Nonrandomized retrospective study	3	31	32	29/3	22/9	53.29 ± 7.55	54.18 ± 9.87	12.77 ± 7.85	13.62 ± 9.21
Zhang et al. (2016) [26]	China	Nonrandomized retrospective study	1, 2	22	27	11/12	13/14	48.6 ± 8.1	52.7 ± 8.3	24	24

**Table 2:** Newcastle-Ottawa Scale evaluation of observational studies.

Methodological quality assessment for inclusion in observational studies (score)									
Research	Study population selection				Intergroup comparison (2 points)	Measurement of exposure factors			Total (9 points)
	A (1 point)	B (1 point)	C (1 point)	D (1 point)		E (1 point)	F (1 point)	G (1 point)	
Alimi, 2016	1	1	1	0	2	1	1	0	7
Li, 2017	1	1	1	0	2	0	1	0	6
Liu, 2016	1	1	1	0	2	1	1	0	7
Cho, 2015	1	1	1	1	2	1	1	1	9
Shi, 2016	1	1	1	1	2	0	1	0	7
Sun, 2020	1	1	1	1	2	1	1	0	8
Wang, 2014	1	1	1	1	2	1	1	0	8
Yang, 2015	1	1	1	0	2	0	1	0	6
Yong, 2018	1	1	1	0	2	1	1	1	8
Yun, 2016	1	1	1	0	2	0	1	0	6
Zhang, 2016	1	1	1	1	2	1	0	0	7

A, case determination being appropriate; B, case representation; C, selection of control; D, determination of control; E, determination of exposure factors; F, determination of case and control exposure factors being the same; G, response rate.

**Table 3:** Meta-analysis results of included studies.

Research projects		Number of studies	Sample size			Results			Heterogeneity *P*-values (*I*^2^)	Statistical methodology
			Total	Zero-P	PC	*P*-value	OR/WMD	CI 95 per cent		
Cervical curvature	Preoperative	5	321	150	171	0.69	−0.23	−1.38, 0.92	0.72 (0%)	WMD (IV, fixed)
	Postoperative	5	321	150	171	0.59	−0.38	−1.77, 1.01	0.85(0%)	WMD (IV, fixed)
Intervertebral fusion rate		6	383	184	199	0.38	0.76	0.27, 2.48	0.97(0%)	OR (M-H, fixed)
DHI	Preoperative	5	380	181	199	0.65	−0.04	−0.14, 0.22	0.66(0%)	WMD (IV, fixed)
	Postoperative	5	380	181	199	0.675	0.06	−0.22, 0.34	0.03(63%)	WMD (IV, random)
Dysphagia		13	904	458	446	<0.00001	0.40	0.28, 0.58	0.62(0%)	OR (M-H, fixed)
ALOD		2	133	63	70	0.001	0.09	0.02, 0.39	0.57(0%)	OR (M-H, fixed)
ASD		6	440	219	221	0.02	0.42	0.20, 0.86	0.20(32%)	OR (M-H, fixed)
Sinking rate of the cage		5	448	221	227	0.50	1.41	0.52, 3.82	0.09(51%)	OR (M-H, random)
Screw loosening		3	326	164	162	0.0009	0.20	0.08, 0.52	0.25(28%)	OR (M-H, fixed)

## Data Availability

The data used in this study are all included in this paper and open to all readers.
